# Cross‐Cultural Adaptation and Psychometric Evaluation of the Persian Version of the ADL Taxonomy Questionnaire in Persons With Stroke: A Rasch Analysis

**DOI:** 10.1155/oti/6677040

**Published:** 2026-02-26

**Authors:** Esmail Sadeghi, Narges Shafaroodi, Mandana Fallahpour, Jamileh Abolghasemi, Afsoon Hassani Mehraban

**Affiliations:** ^1^ Department of Occupational Therapy, School of Rehabilitation Sciences, Iran University of Medical Sciences, Tehran, Iran, iums.ac.ir; ^2^ Division of Occupational Therapy, Department of Neurobiology, Care Sciences and Society, Karolinska Institutet, Stockholm, Sweden, ki.se; ^3^ Department of Biostatistics, School of Public Health, Iran University of Medical Sciences, Tehran, Iran, iums.ac.ir

**Keywords:** activities of daily living, ADL Taxonomy, cross-cultural adaptation, occupational therapy, psychometric evaluation, Rasch analysis, stroke

## Abstract

**Introduction:**

Cultural adaptation of assessment tools is essential to ensure their accuracy and relevance across different populations.

**Objectives:**

The study focused on cultural adaptation and psychometric evaluation of the Persian ADL Taxonomy for assessing daily activities in persons with stroke.

**Data Collection Location:**

Data were collected in a clinical setting and a university hospital in Tehran.

**Methods:**

The ADL Taxonomy was translated into Persian in accordance with standard guidelines. Face validity was tested with 20 stroke patients and convergent validity with 104. Validity and test–retest reliability were analyzed using SPSS, Spearman correlation with MBI scores, and Pearson correlation for reliability(*p* < 0.05). Rasch analysis was performed in R (Version 4.4.1) using the eRm and TAM packages with the 1PL model for dichotomous data, analyzing each of the 46 actions separately. Item difficulty, standard errors, model fit (Infit MnSq ≤ 1.3; *t* = −2 to +2), and discrimination indices (≥ 0.30) were calculated. Person ability estimates were obtained using the EAP method, with PSI (≥ 2.0) and PR (≥ 0.80) calculated and raw scores examined to assess the scale′s reliability, discriminative ability, and potential ceiling or floor effects.

**Results:**

Face validity confirmed item clarity, with minor edits. Impact scores ranged from 2.70 to 5.00. Content validity indices were high (CVR = 0.54–1.0; $\begin{array}{c} \text{I}–\text{CVI}=0.841.0– \end{array}$; $\begin{array}{c} \text{S}–\text{CVI}/\text{UA}=91.3\% \end{array}$; $\begin{array}{c} \text{S}–\text{CVI}/\text{Ave}=0.99 \end{array}$; modified kappa = 0.84–1.0). Convergent validity showed a strong correlation with the MBI (*r* = 0.89, *p* < 0.001), and test–retest reliability was excellent (ICC = 0.98). Rasch analysis of items showed a wide range of difficulty (−4.77 to 6.61 logits) with acceptable measurement precision, generally good model fit, and adequate discrimination for most items, although a few (e.g., communication and transportation and traveling) displayed misfit and low discriminatory power. Person ability estimates ranged widely (2–46, median = 34, mean = 32.05, Q1 = 25.75, Q3 = 42), with a person separation index of 3.95 and person reliability of 0.94.

**Conclusions:**

The Persian version of the ADL Taxonomy demonstrated excellent validity and reliability, with most items showing good fit and discrimination. The scale reliably distinguished between five levels of ability, and no ceiling or floor effects were observed in the Rasch analysis, supporting its use for assessing daily activities in Persian‐speaking stroke patients.

## 1. Introduction

Stroke is one of the leading causes of disability worldwide and is associated with substantial physical, cognitive, and emotional consequences for affected individuals [1]. According to global statistics, over 12 million people experience a stroke each year, making it a major public health concern. The global prevalence of stroke worldwide has significantly increased between 1990 and 2021, largely due to reduced mortality and improved clinical interventions [2].

The incidence of stroke in Iran is higher than in Western countries. Based on provincial and regional studies, the annual incidence is estimated to be 149–113 per 100,000 people of all ages and more than 500 cases per 100,000 individuals aged over 45 years old [3, 4]. Based on a systematic review and meta‐analysis published in 2021, the overall prevalence of stroke in Iran is estimated to be 3.7% of the population [5]. Although stroke‐related mortality has declined, the number of people living with the long‐term consequences of stroke has increased due to population growth and aging. The increasing number of persons with stroke creates a higher demand for rehabilitation services [6].

Participation, defined as involvement in life situations according to the International Classification of Functioning, Disability, and Health (ICF) (WHO, 2001), is essential for well‐being and quality of life. Previous research in Iran shows that persons with stroke experience substantial restrictions in participation in daily activities [7–9], impacting their life satisfaction [8]. Enhancing participation in daily activities is a central goal of occupational therapy [10]. Assessment of restrictions in participation in daily activities is therefore crucial for rehabilitation, to understand the impact of stroke on an individual′s life and identify their specific needs for intervention [11]. Reliable, culturally relevant assessment tools are therefore essential for documenting outcomes in both clinical and research settings [12].

In Iran, commonly used OT assessment tools, such as the Canadian Occupational Performance Measure (COPM) [13, 14] and the Modified Barthel Index (MBI) [15, 16], have limitations. COPM reflects self‐perceived performance, which can vary widely, while MBI lacks a complete assessment of both personal activities of daily living (PADL) and instrumental activities of daily living (IADL). The ADL Taxonomy offers a comprehensive alternative, assessing various daily activities and their related actions. This instrument, which was originally developed in Sweden in the 1990s, categorizes daily activities into 12 groups, with specific actions organized hierarchically by complexity [17, 18]. It includes both PADL and IADL, such as eating, grooming, and household tasks [12]. This instrument uses a flexible scoring system to assess levels of ability and independence. It has several adapted versions, including the original version, as well as versions for children, individuals with visual impairments, and those with mental disabilities [19]. Previous research has utilized this instrument across various conditions, such as Parkinson′s disease [20] and psychiatric disabilities [21, 22]. ADL Taxonomy can be administered via interviews, observation, and self‐reports across environments like hospitals or homes [17].

The ADL Taxonomy has been translated into Danish, Norwegian, and Thai [19]. Psychometric evaluation of the original ADL Taxonomy across various populations confirms its content validity and adequate reliability [23]. Although the instrument has been translated and tested into three languages other than Swedish [19], a Persian version of this tool is needed. Given cultural differences, adapting the ADL Taxonomy for Iranian occupational therapists could provide a more relevant and comprehensive assessment of ADL performance, especially among persons with stroke. Therefore, the aim of this study was to translate, cross‐culturally adapt, and evaluate the validity and reliability of the Persian version of the ADL Taxonomy questionnaire to use among persons with stroke.

## 2. Material and Methods

### 2.1. Design

**Figure 1:**
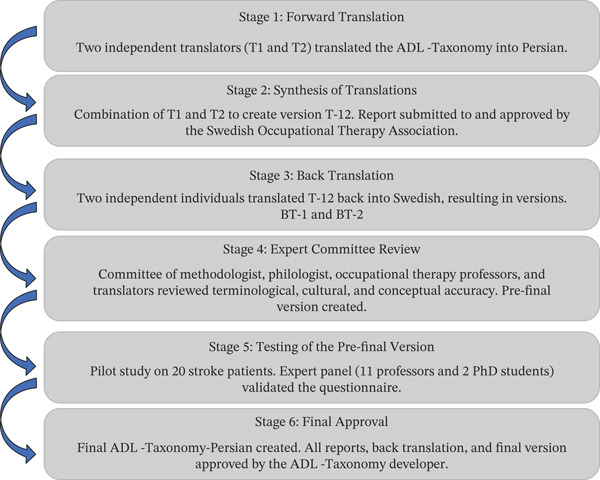
Graphic representation of the translation and cross‐cultural adaptation process used in the study. Stepwise process including forward translation, synthesis, backtranslation, expert review, pretesting, and finalization to ensure equivalence with the original instrument.

This cross‐sectional study comprised two phases: (i) cross‐cultural adaptation and (ii) psychometric evaluation, based on the latest ADL Taxonomy manual [19]. In the adaptation phase, after obtaining permission from the developers and the Swedish Association of Occupational Therapists, the Swedish version was translated into Persian following established guidelines [24]. This process included forward translation, synthesis, backtranslation, expert review, and pretesting with the target population (Figure 1). All procedures were conducted in coordination with the original developers to ensure fidelity. In the psychometric phase, the adapted version was administered to individuals with stroke to evaluate content and convergent validity, as well as test–retest reliability using the intraclass correlation coefficient (ICC). Additionally, Rasch analysis was performed to assess model fit, item difficulty, and item discrimination.

### 2.2. Participants

Participants were recruited using convenience sampling from the neurology department of a university hospital and a rehabilitation clinic in Tehran. The inclusion criteria were (i) stroke diagnosis, (ii) proficiency in the Persian language, (iii) residence in Tehran, (iv) absence of other disability‐related disorders, and (v) living in noninstitutional community settings. Ethical approval was granted by the Ethics Committee of Iran University of Medical Sciences (No. IR.IUMS.REC.1402.078), and written informed consent was obtained from all participants before their inclusion in the study. Study information was provided to 116 individuals, of whom 104 provided informed consent and agreed to participate.

### 2.3. Data Collection

Demographic information (age, gender, affected side, and time since stroke) was collected through interviews. Functional status was assessed using the backtranslated ADL Taxonomy [17] and the MBI [15].

### 2.4. ADL Taxonomy (Original Version)

The ADL Taxonomy [17] assesses performance in personal and instrumental ADLs, comprising 12 activities encompassing 46 related actions. These activities include eating, drinking, mobility, toileting, dressing, personal hygiene, grooming, communication, transportation, cooking, shopping, cleaning, and washing [19]. Each activity includes three to six actions, ranked from simple to complex. Actions are evaluated as “not applicable,” “does,” or “does not,” “wants to do” or “does not want to do,” and “can do” or “cannot do.” The results are recorded in a table with checkboxes or in a spider chart (ADL Taxonomy circle). The original ADL Taxonomy has demonstrated content and construct validity through expert consensus and theoretical grounding in occupational therapy concepts [17].

### 2.5. MBI

The MBI [15] is a widely used tool to measure functional independence in individuals, particularly those recovering from stroke or neurological diseases. It evaluates ten ADLs, scoring each activity on a 5‐point scale, with higher scores indicating greater independence. The total score ranges from 0 to 100. The Persian version of the MBI has shown excellent reliability (*α* = 0.955) and strong construct validity (83.2% variance explained) in Iranian geriatric stroke inpatients [25].

### 2.6. Translation and Cross‐Cultural Adaptation

The translation and cross‐cultural adaptation of the ADL Taxonomy into Persian followed a structured, multiphase process in accordance with international guidelines. Initially, two independent forward translations (T1 and T2) were performed by bilingual translators. These two versions were then synthesized into a unified Persian draft (T‐12) through discussion and consensus. To evaluate the conceptual accuracy, the T‐12 version was backtranslated into Swedish by two new independent translators, producing two backtranslated versions (BT‐1 and BT‐2). A multidisciplinary expert committee, consisting of specialists in occupational therapy, linguistics, methodology, and translation, reviewed all translated versions to ensure semantic, cultural, and conceptual equivalence. Based on their evaluation, a prefinal version was developed. This version was pilot‐tested with 20 persons with stroke to evaluate clarity and comprehensibility, and the expert panel provided validation. After final revisions, all materials, including translation reports and backtranslations, were reviewed and approved by the original developer of the ADL Taxonomy, resulting in the finalized Persian version. Figure 1 presents an overview of the translation and cross‐cultural adaptation process employed in this study.

### 2.7. Data Analysis

The study examined multiple aspects of validity and reliability of the Persian version of ADL Taxonomy in a Persian sample of persons with stroke as follows.

Convergent validity and test–retest reliability were analyzed using SPSS (Version 27). Normal distribution of the data was assessed with the Kolmogorov–Smirnov test (*p* > 0.05). Spearman correlation evaluated the association between ADL Taxonomy and MBI scores, while Pearson correlation assessed test–retest reliability. A significance level of *p* < 0.05 was applied throughout.

Rasch analysis was performed using R software (Version 4.4.1, R Foundation for Statistical Computing, Vienna, Austria) with the eRm and TAM packages. Rasch analysis was applied using the simple Rasch model (1PL) for dichotomous data (“does/does not”), with each of the 46 actions analyzed separately as independent scales. Item difficulty (logits) and standard errors (SEs) were estimated, with negative, near‐zero, and positive logits representing easy, moderate, and difficult items. Model fit was evaluated using Infit MnSq (mean square residual) (≤ 1.3) and *t* values (−2 to +2), with MnSq used as the main criterion due to the effect of sample size on *t* values. The discrimination index was also calculated, with values ≥ 0.30 considered acceptable. Person ability estimates were obtained using the expected a posteriori (EAP) method, and the person separation index (PSI) and person reliability (PR) were calculated to assess the scale′s ability to discriminate between participants with different levels of ADL performance. Participants with extreme response patterns, for whom SEs could not be defined, were excluded from these calculations. The distribution of raw scores was inspected through descriptive statistics to evaluate potential ceiling or floor effects.

### 2.8. Face Validity

Face validity refers to a subjective assessment of whether a test or measurement appears to measure what it claims to measure, based on whether the items in each domain are perceived as reasonable, appropriate, and relevant by individuals who use the measure in their daily lives [26]. For qualitative face validity, 20 persons with stroke were interviewed by occupational therapists who completed the prefinal Persian ADL Taxonomy, evaluating item difficulty, relevance, and clarity [27]. For quantitative analysis, 13 occupational therapists rated each item on a 5‐point Likert scale. Impact scores (ISs) above 1.5 were considered acceptable [28].

### 2.9. Content Validity

Content validity refers to how well a tool′s content reflects the structure it is intended to measure, and its assessment requires expert judgment [29]. In this study, content validity was evaluated using the content validity ratio (CVR) and content validity index (CVI) [30]. Thirteen experts in occupational therapy, including eleven faculty members with doctoral degrees and two PhD candidates, rated each item as essential, useful, or not essential. Items with a CVR above 0.54 were retained [31]. For CVI, experts assessed relevance, clarity, simplicity, and specificity. Items with a CVI ≥ 0.79 were deemed acceptable [32]. The CVI was calculated for individual item‐content validity ratio (I‐CVI) and for the entire scale using two methods of (i) scale‐content validity ratio based on universal agreement (S‐CVI/UA) (≥ 0.80) and (ii) scale‐content validity ratio based on the average (S‐CVI/Ave) (≥ 0.90) [30, 33]. Kappa statistics were used to assess agreement, with values above 0.74 indicating excellent agreement [34, 35].

### 2.10. Convergent Validity

Tools chosen for convergent validity assessments are therefore selected based on their conceptual similarity to the target construct, supporting the evaluation of whether the instrument accurately measures what it intends to [36].

In this study, convergent validity was assessed by examining the correlation between the total scores of the Persian ADL Taxonomy and the total scores of the MBI. The ADL Taxonomy total score was derived as the percentage of applicable actions (from a total of 46) that the individual could perform independently, resulting in a score ranging from 0 to 100. This scoring approach was approved by the original developer of the ADL Taxonomy. Convergent validity was assessed based on the hypothesis that there would be a strong positive correlation between the measures due to their conceptual similarity. A correlation coefficient ≥ 0.5 was considered indicative of adequate convergent validity [36].

### 2.11. Test–Retest Reliability

Test–retest reliability refers to the consistency of repeated measurements, indicating the stability of patients on the construct being measured [29]. Test–retest reliability was measured using the ICC, with values > 0.70 indicating acceptable reliability [37]. Participants were retested 10–15 days after the initial assessment under similar conditions, without prior knowledge of their initial responses [38].

### 2.12. Rasch Analysis (Construct Validity)

To assess construct validity and to examine the hierarchical order of actions within each activity, the ADL Taxonomy questionnaire was analyzed using the Rasch measurement model [39]. Analyses were conducted separately for each of the 46 actions, meaning that the ADL Taxonomy was not treated as a single unified scale. Instead, each action was analyzed as an independent scale with its own specific items. In this process, only binary response options (“does”/“does not”) were used for each action [40, 41]. This approach aligns with the theoretical structure of the ADL Taxonomy, which conceptualizes activities of daily living as discrete, observable actions rather than indicators of a single latent trait [17]. Applying Rasch analysis at the level of individual actions allows for precise evaluation of item functioning, person ability estimates, and targeting, without imposing assumptions of unidimensionality that are not supported by the instrument′s conceptual framework. This strategy ensures that the measurement approach is consistent with the occupation‐based perspective of the ADL Taxonomy and preserves the interpretability of scores for each action [39].

In the Rasch model, the item difficulty index represents the relative difficulty of an item in distinguishing individuals with different ability levels. This index is expressed in logarithmic units (logits) and reflects the point at which a person with a given ability has a 50% probability of successfully performing the item. Negative values correspond to easier items, values near zero represent items of moderate difficulty, and positive values indicate more difficult items. The SE of the difficulty estimate reflects the precision of the measurement, with lower SE values indicating greater accuracy [42].

For dichotomous data, the simple Rasch model (one‐parameter logistic model, 1PL) was applied. This model assumes that the probability of a correct response to an item is determined solely by the respondent′s ability and the item′s difficulty. Model fit was assessed through MnSq indices and standardized *t* values. Given the study objectives, Infit statistics were emphasized, as they are more sensitive to misfit in the range of individuals′ ability levels and are more strongly related to actual performance [39]. Based on established criteria, Infit MnSq values below 1.3 together with Infit *t* values between −2 and +2 were considered indicative of acceptable fit [43].

The discrimination index was also examined to assess the extent to which each questionnaire item differentiated between respondents with different levels of ability. This index reflects the item′s capacity to distinguish between high‐ and low‐performing individuals. Values (≥ 0.30) were considered indicative of adequate discriminatory power [44].

The PSI, which reflects the scale′s ability to distinguish between participants with different ability levels, and PR, which indicates the consistency of these measurements, were calculated, excluding participants with extreme scores. Raw score distributions were inspected to check for ceiling or floor effects. Generally, PSI values ≥ 2.0 and PR values ≥ 0.80 are considered acceptable in Rasch analysis [45].

## 3. Results

### 3.1. Demographic Characteristics

This study included 104 patients. The characteristics of the study population are presented in Table 1.

**Table 1:** Demographic data of patients (*n* = 104).

Variable	*M* *e* *a* *n* ± *S* *D*	*N*	%
Gender			
Male		57	54.8
Female		47	45.2
Age (years)			
Male	62.1 ± 12.1		
Female	59.8 ± 11.7		
Time after stroke (years)	4.2 ± 3.2		
Affected side			
Right		53	51
Left		46	44.2
Both		5	4.5

### 3.2. Translation and Cross‐Cultural Adaptation

The final changes made to the items in the Persian version, compared to the original Swedish version, are summarized in Table 2.

**Table 2:** Changes made during the translation and cultural adaptation of the ADL Taxonomy into Persian.

Item	Original (Swedish)	Modified (Persian)	Type of cultural adaptation
Eating and drinking—Third subitem	Preparing/cutting food using a knife and fork	Preparing/cutting food using a spoon and fork	Adapted to reflect common Iranian eating utensils
Going to the toilet—Definition	Refers to general toilet use	Includes both Western‐style and traditional Iranian toilets	Adapted to reflect dual toilet systems in Iranian homes
Grooming—Definition	Caring for other parts of the body	Grooming	Simplified for clarity and cultural alignment
Grooming—Fourth subitem	Manicure	Trimming hand nails (cutting, filing)	Replaced with culturally familiar terminology
Grooming—Fifth subitem	Pedicure	Trimming foot nails (cutting, filing)	Replaced with culturally familiar terminology
Traveling—Definition	Traveling	Transportation and traveling	Broadened to better reflect local understanding
Traveling—Fourth subitem	Cycling/riding a motorcycle	Cycling/driving an electric or gasoline motorcycle	Clarified and adapted to reflect common transportation methods
Washing—Definition	Mangla	Ironing	Replaced an unfamiliar term with a culturally known equivalent

### 3.3. Face Validity

The qualitative face validity assessment indicated that the items were generally clear and understandable. The quantitative assessment, based on the IS ranging from 2.70 to 5.00, indicated that all items were considered important (Table 3). The expert panel confirmed that evaluating aspects such as ambiguity, relevance, terminology, and grammatical clarity was appropriate for the target population.

**Table 3:** Content validity index, modified kappa, and comprehensiveness of instrument dimensions and total instrument (*N* = 13).

Activity	Action	IS	Number giving a rating of 3 or 4 to the relevance of the item	I‐CVI	PC	*K*	Interpretation
1. Eating and drinking							
A1	Eating means bringing food to the mouth and eating	5	13	1	0.00012	1	Excellent
A2	Drinking means bringing a beverage to the mouth and consuming it	5	13	1	0.00012	1	Excellent
A3	Preparing/cutting food means preparing food and drinks and cutting food with a spoon and fork	4.92	13	1	0.00012	1	Excellent
2. Mobility							
A1	Moving oneself in bed means changing positions (rolling over and sitting up)	4.92	13	1	0.00012	1	Excellent
A2	Transferring oneself from the bed to a chair or from one chair to another	5	13	1	0.00012	1	Excellent
A3	Moving between the rooms on one floor	4.92	13	1	0.00012	1	Excellent
A4	Moving between floors via the elevator or stairs	4.86	13	1	0.00012	1	Excellent
A5	Moving in and out of the house	5	13	1	0.00012	1	Excellent
A6	Moving around outside the house	5	13	1	0.00012	1	Excellent
3. Going to the toilet							
A1	Voluntary bowel and bladder control	4.92	13	1	0.00012	1	Excellent
A2	Moving on the toilet and cleaning oneself after defecation and getting up from the toilet	4.92	13	1	0.00012	1	Excellent
A3	Organizing clothes and any hygiene aids such as diapers and wipes and washing hands	4.46	13	1	0.00012	1	Excellent
A4	Going to the bathroom at the right moment when feeling the urge	5	13	1	0.00012	1	Excellent
4. Dressing and undressing							
A1	Taking off clothes	5	13	1	0.00012	1	Excellent
A2	Putting on upper‐body clothing	5	13	1	0.00012	1	Excellent
A3	Putting on lower‐body clothing	5	13	1	0.00012	1	Excellent
A4	Putting on socks/tights and shoes	1	13	1	0.00012	1	Excellent
A5	Taking out the necessary clothes from the closet/pulling out the clothes drawer	3.84	13	1	0.00012	1	Excellent
5. Personal hygiene							
A1	Washing the face and hands	5	13	1	0.00012	1	Excellent
A2	Taking a bath/showering	4.92	13	1	0.00012	1	Excellent
A3	Washing hair	4.86	12 (total number of experts)	1	0.00024	1	Excellent
6. Grooming							
A1	Combing hair/styling hair	4.86	13	1	0.00012	1	Excellent
A2	Brushing teeth	5	13	1	0.00012	1	Excellent
A3	Shaving/doing makeup	4.92	13	1	0.00012	1	Excellent
A4	Trimming hand nail (cutting, filing)	4.92	13	1	0.00012	1	Excellent
A5	Trimming foot nail (cutting, filing)	4.40	13	1	0.00012	1	Excellent
7. Communication							
A1	Making contact/attracting attention	4.86	13	1	0.00012	1	Excellent
A2	Taking part in a conversation	4.92	13	1	0.00012	1	Excellent
A3	Using the telephone	4.86	13	1	0.00012	1	Excellent
A4	Having the ability to read	4.33	13	1	0.00012	1	Excellent
A5	Having the ability to write	4.33	13	1	0.00012	1	Excellent
8. Transportation and traveling							
A1	Traveling/moving by car	4.19	12	0.92	0.0016	0.92	Excellent
A2	Traveling/moving with public transportation	4.86	13	1	0.00012	1	Excellent
A3	Traveling/moving by train, boat, or airplane	3.66	13	1	0.00012	1	Excellent
A4	Cycling/driving on an electric or gasoline motorcycle	2.70	12	0.92	0.0016	0.92	Excellent
A5	Driving/riding a motorcycle	3.66	13	1	0.00012	1	Excellent
9. Cooking							
A1	Preparing a cold meal	4.86	13	1	0.00012	1	Excellent
A2	Heating up liquid or prepared food	4.86	13	1	0.00012	1	Excellent
A3	Preparing/cooking a hot meal	4.92	13	1	0.00012	1	Excellent
10. Shopping							
A1	Planning for shopping (e.g., making a shopping list and shopping online)	4.77	13	1	0.00012	1	Excellent
A2	Doing small shopping at a nearby store	4.92	13	1	0.00012	1	Excellent
A3	Doing bulk or weekly shopping	3.84	11	0.84	0.0094	0.84	Excellent
11. Cleaning							
A1	Performing light and minor daily cleaning	5	13	1	0.00012	1	Excellent
A2	Doing the weekly cleaning	4.92	11	0.84	0.0094	0.84	Excellent
12. Washing							
A1	Washing small clothes by hand or washing machine	4.92	13	1	0.00012	1	Excellent
A2	Washing heavy clothes (like bed sheets) with a washing machine	4.19	13	1	0.00012	1	Excellent

Abbreviations: I‐CVI, item‐level content validity index; IS, impact score; *K*, modified kappa; PC, probability of a chance occurrence.

### 3.4. Content Validity

The CVR values for each item ranged from 0.54 to 1.0, indicating that all items were considered essential or highly relevant by experts. The I‐CVI values ranged from 0.84 to 1.0, showing high relevance and clarity for all items. The S‐CVI/UA value of 91.3% and S‐CVI/Ave of 0.99 reflect strong expert consensus on the importance and coverage of daily life activities. Additionally, the modified kappa values ranged from 0.84 to 1.0, demonstrating a high degree of agreement among experts, further supporting the validity and reliability of the questionnaire (Table 3).

### 3.5. Convergent Validity

The correlation between the total score of the ADL Taxonomy and the total score of the MBI revealed a strong positive correlation (*r* = 0.89, *p* < 0.001). These results indicate a significant and strong correlation, demonstrating the high convergent validity of the ADL Taxonomy. The strong alignment with the MBI, a well‐established performance assessment tool, suggests that both instruments effectively measure the same underlying construct.

### 3.6. Test–Retest Reliability

The test–retest reliability of the Persian version of the ADL Taxonomy was excellent, with an ICC of 0.98 (95% CI: 0.96–0.99), indicating high stability and consistency over time.

### 3.7. Rasch Analysis (Construct Validity)

The findings of the Rasch model analysis for the 46 items of the ADL Taxonomy questionnaire are summarized in Table 4. The task measure (item difficulty) values ranged from −4.77 to 6.61 logits. Items A11^1^ (eating means bringing food to the mouth and eating) and A12 (drinking means bringing a beverage to the mouth and consuming it) represented the easiest tasks, whereas item A84 (cycling/driving on an electric or gasoline motorcycle) (6.61 logits) was among the most difficult. The SEs of the difficulty estimates ranged from 0.29 to 0.80, indicating acceptable measurement precision.

**Table 4:** Task measure (item difficulty), item fit statistics, and discrimination for the Persian version of the ADL Taxonomy questionnaire based on Rasch analysis (*n* = 104).

Item	Task measure	SE	Infit		Discrimination
			MnSq	*t*	
A11	−4.77	0.8	0.4	−1.3	0.4
A12	−4.77	0.8	0.4	−1.3	0.4
A13	−2.23	0.43	1.0	0.2	0.6
A21	−3.80	0.62	1.1	0.3	0.5
A22	−2.23	0.43	0.5	−2.0	0.7
A23	−2.23	0.43	0.5	−2.0	0.7
A24	−1.16	0.35	0.7	−1.7	0.7
A25	−1.04	0.35	0.4	−2.0	0.8
A26	−0.68	0.33	0.4	−2.0	0.8
A31	−2.43	0.45	0.7	−1.2	0.6
A32	−2.23	0.43	0.5	−1.9	0.7
A33	−0.68	0.33	1.2	1.3	0.6
A34	−2.88	0.49	0.6	−1.4	0.6
A41	−1.88	0.40	0.8	−0.9	0.7
A42	−1.29	0.36	0.9	−0.4	0.7
A43	−0.68	0.33	0.9	−0.5	0.7
A44	−0.36	0.32	1.1	0.8	0.6
A45	−0.36	0.32	0.5	−2.0	0.8
A51	−3.15	0.53	0.4	−2.0	0.7
A52	0.32	0.3	0.9	−0.9	0.7
A53	−1.57	0.38	0.8	−0.7	0.7
A61	−1.88	0.40	0.8	−0.6	0.7
A62	−0.46	0.32	0.7	−1.5	0.7
A63	0.50	0.30	0.6	−1.9	0.8
A64	2.55	0.30	1.3	1.6	0.6
A65	2.64	0.30	0.7	−1.7	0.6
A71 ^∗^	−2.23	0.43	**2.0**	**2.9**	0.3
A72 ^∗^	−2.23	0.43	**1.8**	**2.6**	0.4
A73	−0.46	0.32	1.2	1.1	0.6
A74 ^∗^	0.32	0.30	**2.4**	**6.2**	**0.2** ^∗∗^
A75	2.04	0.29	1.2	1.2	0.6
A81 ^∗^	−0.46	0.32	**2.1**	**4.8**	0.3
A82	1.61	0.29	1.2	1.0	0.7
A83 ^∗^	−0.15	0.31	**1.5**	**2.5**	0.5
A84	6.61	0.47	1.3	1.6	**0.0** ^∗∗^
A85	5.74	0.39	1.3	1.9	0.3
A91	0.23	0.30	0.7	−1.9	0.8
A92	1.20	0.29	0.6	−1.8	0.8
A93	2.55	0.30	0.8	−1.3	0.7
A101	2.81	0.30	0.7	−2.0	0.7
A102	3.17	0.31	0.5	−1.7	0.7
A103	4.01	0.32	0.5	−2.0	0.6
A111	2.12	0.29	0.8	−1.1	0.7
A112	3.54	0.32	0.7	−1.9	0.6
A121	2.46	0.30	1.0	0.2	0.6
A122	3.92	0.32	0.6	−2.0	0.6

*Note:* Axx = activity x, action x (e.g., A11 = Activity 1, Action 1; A121 = Activity 12, Action 1).

Abbreviations: MnSq, mean square of the residuals; SE, standard error.

^∗^Misfitting task in the Rasch analysis (Infit MnSq ≥ 1.3 or *t* values outside −2 to +2).

^∗∗^Items with insufficient discriminatory power (discrimination < 0.30).

Regarding model fit, most items demonstrated Infit MnSq values within the acceptable threshold (≤ 1.3), and Infit *t* values were largely within the range of −2 to +2. However, several items showed misfit with the model, particularly A71 (making contact/attracting attention) (Infit MnSq = 2.0, *t* = 2.9), A72 (taking part in a conversation) (1.8, *t* = 2.6), A74 (having the ability to read) (2.4, *t* = 6.2), A81 (traveling/moving by car) (2.1, *t* = 4.8), and A83 (traveling/moving by train, boat, or airplane) (1.5, *t* = 2.5).

For the discrimination index, most items reached acceptable levels (≥ 0.30). Nonetheless, items such as A74 (having the ability to read) (0.20) and A84 (cycling/driving on an electric or gasoline motorcycle) (0.00) exhibited insufficient discriminatory ability.

Person ability estimates demonstrated a broad distribution. In the initial Rasch analysis of the Persian ADL Taxonomy, raw scores ranged from 2 to 46 (median = 34, mean = 32.05, first quartile = 25.75, third quartile = 42), with a PSI of 3.95 and PR of 0.94. The PSI was 3.95, and PR was 0.94, indicating that the scale could reliably distinguish approximately five statistically distinct levels of ADL ability. No ceiling or floor effects were observed in the distribution of raw scores. Following the identification and removal of items exhibiting misfit or low discrimination, the revised model showed raw scores ranging from 0 to 40 (median = 30, mean = 27.93, first quartile = 22, third quartile = 37). In the reduced model, the PSI increased to 4.17 and PR to 0.95.

## 4. Discussion

The ADL Taxonomy is a flexible tool that has been used to evaluate participation in activities of daily living across various populations, including individuals with Parkinson′s disease [20, 46–48], severe psychiatric disabilities [49], essential tremor [21, 22, 50], osteoarthritis [51], congenital heart disease [52], and individuals with brain injuries [53–55]. It has been widely adopted by occupational therapists in countries such as Sweden, Denmark, and Norway and has been translated into multiple languages, such as Finnish, Thai, and German [19].

The findings of this study are consistent with previous research and provide additional psychometric support for both the original and adapted versions of the ADL Taxonomy. The original instrument demonstrated acceptable content validity [17], construct validity through its ordered categorical structure, and discriminatory validity across clinical groups [18]. Adapted versions for populations with mental disorders [41] and acquired brain injuries [12] also showed strong psychometric properties based on Rasch analysis. In the study by Wæhrens and Fisher (2009), excellent internal consistency and construct validity of the ADL Taxonomy were demonstrated. Rasch analysis revealed high person and item separation indices of 5.94 (reliability = 0.97) and 6.35 (reliability = 0.98), respectively. Furthermore, the items were shown to define a unidimensional construct of ADL ability, indicating that the scale reliably measures a coherent underlying trait, even when some misfitting items are retained [12]. Although test–retest reliability has not been formally assessed, interrater agreement has ranged from 94% to 100% [19], indicating strong observational reliability.

The translation and cross‐cultural adaptation followed established [24], ensuring process conceptual equivalence and cultural relevance. Close collaboration between Iranian and Swedish experts helped resolve cultural differences, maintaining the adapted version′s fidelity to the original instrument while making it contextually appropriate for Iranian users. A key strength of this study was the cultural adaptation of several ADL Taxonomy items to better reflect Iranian daily living practices. For example, in the “eating and drinking” item, the original “knife and fork” was replaced with “spoon and fork” to align with common Iranian eating utensils. Similarly, unfamiliar terms such as “Mangla” were replaced with “ironing,” a more common term. These modifications improved clarity and cultural relevance while maintaining the original meaning of the items.

Face validity assessment with persons with stroke indicated that the items were generally clear and understandable, with only minor revisions required. As face validity has not been explicitly addressed in previous ADL Taxonomy studies, its inclusion represents an important contribution to the current psychometric evaluation.

The content validity of the original ADL Taxonomy was established through expert consensus and empirical evidence derived from its clinical implementation. Developed within a defined conceptual framework, it is organized into three hierarchical levels: occupational forms, activities, and actions [17]. An expert panel of occupational therapists reviewed the initial version, with their feedback informing iterative revisions and subsequent clinical testing to ensure practical relevance [17, 18]. In the present study, strong content validity was confirmed, with high CVR, I‐CVI, and S‐CVI values. The S‐CVI/UA was 91.3%, and the S‐CVI/Ave was 0.99, exceeding the recommended threshold of 0.90, indicating excellent expert agreement on item relevance. Modified kappa values ranged from 0.84 to 1.0, reflecting a very high level of agreement beyond chance and supporting the clarity and appropriateness of the questionnaire items.

In this study, convergent validity analysis revealed a strong positive correlation between the Persian ADL Taxonomy and the MBI, indicating that the adapted version effectively captures key constructs of daily functioning. Similar findings have been reported in the original Swedish version, where moderate to high correlations were observed between several ADL domains and the General Motor Function Scale (GMF) [56].

Although test–retest reliability had not been formally assessed, the ADL Taxonomy demonstrates excellent interrater reliability [19]. Wæhrens and Fisher (2009) showed that the ADL Taxonomy has excellent internal consistency and strong reliability based on Rasch analysis [12]. In the present study, excellent test–retest reliability (ICC = 0.98) and strong internal consistency were observed, indicating that the Persian version produces stable and reliable results in both clinical and research contexts.

The difficulty estimates of the questionnaire items showed that the ADL Taxonomy covers a wide spectrum of activities, ranging from simple ones (e.g., eating and drinking) to more complex ones (e.g., social interactions or advanced motor skills). This wide gradient enables assessment across varying levels of functional ability, allowing detection of both severe limitations and subtle performance differences among higher‐functioning individuals through more difficult items. The SE values further indicated that item difficulty estimates were obtained with reasonable precision.

Construct validity findings support the original ADL Taxonomy as a valid instrument for documenting daily activity performance in line with its theoretical framework [17]. Additionally, Wæhrens and Fisher (2009) demonstrated the construct validity of the ADL Taxonomy, showing through Rasch analysis that its items measure a single, coherent dimension of ADL ability, maintaining reliability despite some misfitting items [12]. Similarly, Rasch analysis of an adapted version for individuals with mental disorders demonstrated acceptable validity for most activities, while retaining certain clinically relevant but inconsistent items [41]. The decision to analyze each ADL action as an independent dichotomous scale is conceptually justified. The ADL Taxonomy treats each action as a distinct component of daily functioning, which may vary independently across individuals depending on task demands, environmental context, and personal skills [17]. Aggregating actions into a single scale could obscure clinically meaningful differences in performance and reduce the interpretability of the results. Analyzing actions separately preserves the occupation‐based perspective of the ADL Taxonomy and enhances sensitivity to clinically relevant changes, which is essential for guiding targeted occupational therapy interventions and monitoring progress over time [39, 57].

The findings of the Rasch analysis from the Persian version indicated that the majority of items had Infit MnSq and Infit *t* values within acceptable ranges, suggesting good overall model fit. However, several items demonstrated poor fit. For instance, social interaction items (A71 and A72) may be influenced by factors beyond motor or basic daily living abilities, such as personality, communication skills, or cultural norms. The reading ability item (A74) appeared to reflect literacy and educational background more than functional capacity, limiting its clinical utility in stroke populations. Similarly, “car travel” (A81) and “long‐distance travel by train, ship, or plane” (A83) may lack relevance for many participants due to cultural, economic, or age‐related factors, which likely explain their poor fit. These findings highlight the need for cautious interpretation of such items and suggest potential directions for future refinement.

In terms of discriminant validity, previous studies on the original ADL Taxonomy confirmed its ability to differentiate between groups and ability levels [40]. The present study similarly showed that most items demonstrated adequate discriminatory power; however, items A74 (reading ability) and A84 (riding a motorcycle or bicycle) did not. These limitations likely reflect cultural relevance and age‐related participation patterns rather than deficiencies in the underlying construct. Accordingly, such items should be interpreted with caution in both clinical and research applications. These findings suggest that in clinical and research applications, such items should be carefully reviewed or interpreted with caution, as cultural, age‐related, or functional irrelevance could compromise the overall validity of the instrument.

Given the sensitivity of participation‐based measures to environmental context, these findings likely reflect urban facilitation rather than weaknesses in the construct being measured [39]. Therefore, although the overall psychometric properties of the Persian version of the ADL Taxonomy were supported, caution is warranted when generalizing these findings to nonurban populations or individuals with lower literacy levels, and further validation in more diverse socioeconomic and geographic contexts is recommended.

Wæhrens and Fisher (2009) reported high PSI and PR for the ADL Taxonomy [12]. Consistently, the Persian version also demonstrated high PSI and PR, effectively distinguishing multiple levels of daily living performance in individuals with stroke. Its ability to differentiate around five performance strata exceeds minimum psychometric standards and supports sensitivity to both broad and subtle functional changes, making it particularly useful for tracking rehabilitation progress [58].

The present study found that the Persian version of the ADL Taxonomy, consistent with the original version reported by Wæhrens and Fisher (2009) [12], showed no floor or ceiling effects in the distribution of raw scores. In contrast, adapted versions of this instrument used in populations with mental disorders [41] have reported ceiling effects for some items, with no floor effects described. The absence of floor and ceiling effects is an important psychometric strength, indicating that the scale can discriminate across the full range of functional abilities in individuals with stroke. This property is particularly relevant for longitudinal studies and occupation‐based interventions, as it enhances responsiveness and reduces the risk of failing to detect meaningful change over time [59]. Accordingly, this characteristic supports the utility of the instrument in both clinical practice and research, particularly for monitoring occupational therapy outcomes.

A brief sensitivity analysis was conducted by comparing the Rasch model before and after the removal of misfitting or low‐discrimination items. The results demonstrated that PSI and PR were stable or improved following item reduction. The observed improvement in these indices suggests that excluding problematic items did not compromise—and in fact enhanced—the scale′s ability to distinguish between multiple strata of person ability. Moreover, although the range of raw scores narrowed slightly in the reduced model, the distribution of scores remained well spread across the scale, indicating preserved construct coverage. These findings support the robustness of the measurement model and suggest that the main results are not sensitive to the inclusion of problematic items. We recommend retaining all items to preserve content validity and clinical comprehensiveness, ensuring that all clinically important aspects of daily living are evaluated. This approach is consistent with the methodology of the ADL Taxonomy adapted version for individuals with mental disorders, in which items exhibiting misfit were retained owing to their clinical significance [41]. Problematic items can be refined in future studies to improve fit and discrimination. Exclusion of misfitting items should be considered only in strictly psychometric research, while all items should be retained for clinical use.

### 4.1. Clinical and Research Applications

The Persian version of the ADL Taxonomy questionnaire, demonstrating promising psychometric properties, holds significant potential as a valuable tool for both clinical practice and research settings. For clinicians, it provides a reliable and culturally relevant means to assess participation in daily activities among Persian‐speaking persons with stroke, enabling more accurate evaluation of patients′ functional abilities. This can help tailor individualized rehabilitation plans, monitor patient progress over time, and identify specific areas requiring intervention. For researchers, the questionnaire offers a standardized and validated instrument that facilitates the collection of consistent data on daily living activities within this population. Its use can contribute to the advancement of evidence‐based rehabilitation approaches, cross‐cultural comparisons, and the development of targeted therapeutic strategies. Overall, the tool bridges a critical gap in the assessment resources available for Persian‐speaking persons with stroke, enhancing both patient care and scientific inquiry in occupational therapy and rehabilitation medicine. Additionally, Rasch analysis results highlight the need for cautious interpretation of certain items, informing potential refinements for future applications.

### 4.2. Limitations

Despite supporting the validity and reliability of the Persian ADL Taxonomy, several limitations should be noted. Participants were recruited through convenience sampling from only two clinical centers in Tehran, which may limit generalizability to other geographic regions, particularly rural areas, or populations with diverse ethnic and cultural backgrounds. Individuals with severe cognitive or speech impairments were excluded. Consequently, the psychometric evaluation of the Persian version of the ADL Taxonomy mainly reflects the performance of individuals with mild to moderate poststroke impairments. This sampling approach may have limited representation at the lower end of the ability continuum, potentially affecting item difficulty and discrimination, especially for more basic activities. The cross‐sectional design captures only a single time point, and some items, especially those related to communication, reading, and transportation, showed misfit or low discrimination in Rasch analysis, likely influenced by cultural or contextual factors. Additionally, while Rasch analysis was performed, the absence of further advanced psychometric evaluations, such as confirmatory factor analysis or differential item functioning (DIF), limits deeper insights into the instrument′s dimensional structure. The primary reason for this is the sample size limitation, as CFA requires larger samples and DIF analysis needs sufficient subgroup representation to detect meaningful item bias.

Future studies should include larger and more diverse samples to enable complementary construct validity analyses, such as CFA and DIF. This would allow for a more robust evaluation of the Persian ADL Taxonomy, examine item functioning across subgroups, and enhance generalizability to broader stroke populations. Including participants from rural areas and varied socioeconomic backgrounds would further clarify environmental influences on item performance.

## 5. Conclusion

This study demonstrates the rigorous cultural adaptation and comprehensive psychometric evaluation of the Persian version of the ADL Taxonomy questionnaire, confirming its validity and reliability. The Persian version of this tool is appropriate and reliable for assessing daily living activities in Persian‐speaking stroke survivors and can support functional evaluations and the design of targeted rehabilitation interventions in both clinical and research settings. Rasch analysis showed that the questionnaire covers a wide range of task difficulty and that most items demonstrate acceptable measurement precision, model fit, and discrimination. Although a few items, particularly those related to communication, reading, and transportation, exhibited misfit or low discriminatory power, sensitivity analysis indicated that their removal does not compromise the overall measurement properties. Overall, the Persian ADL Taxonomy is a valid, reliable, and clinically meaningful instrument that provides robust measurement across a wide spectrum of daily functioning and offers practical guidance for the interpretation and refinement of specific items.

## Author Contributions

All authors have contributed to the study concept, design, and analysis of the data.

## Funding

No funding was received for this manuscript.

## Disclosure

All authors have read and approved the final manuscript.

## Conflicts of Interest

The authors declare no conflicts of interest.

## Endnotes

^1^In this study, ADL Taxonomy items were coded as A[activity number][action number]; for example, A11 = Activity 1, Action 1, and A121 = Activity 12, Action 1.

## Data Availability

The data that support the findings of this study are available on request from the corresponding author. The data are not publicly available due to privacy or ethical restrictions.
